# Charge and spin transport in mesoscopic superconductors

**DOI:** 10.3762/bjnano.5.18

**Published:** 2014-02-17

**Authors:** M J Wolf, F Hübler, S Kolenda, D Beckmann

**Affiliations:** 1 Karlsruher Institut für Technologie (KIT), Institut für Nanotechnologie, P.O. Box 3640, D-72021 Karlsruhe, Germany

**Keywords:** spintronics, superconductor–ferromagnet hybrids

## Abstract

**Background:** Non-equilibrium charge transport in superconductors has been investigated intensely in the 1970s and 1980s, mostly in the vicinity of the critical temperature. Much less attention has been paid to low temperatures and the role of the quasiparticle spin.

**Results:** We report here on nonlocal transport in superconductor hybrid structures at very low temperatures. By comparing the nonlocal conductance obtained by using ferromagnetic and normal-metal detectors, we discriminate charge and spin degrees of freedom. We observe spin injection and long-range transport of pure, chargeless spin currents in the regime of large Zeeman splitting. We elucidate charge and spin transport by comparison to theoretical models.

**Conclusion:** The observed long-range chargeless spin transport opens a new path to manipulate and utilize the quasiparticle spin in superconductor nanostructures.

## Introduction

The investigation of spin-polarized transport in hybrid structures was pioneered in the 1970s with the discovery of spin-dependent tunneling into thin-film superconductors with a large Zeeman splitting by Tedrow and Meservey [1–2]. While much of the related basic physics such as tunneling magnetoresistance (TMR) [3] and non-equilibrium spin injection [4] was observed subsequently, spin-polarized transport did not attract much attention until the discovery of the giant magnetoresistance (GMR) [5–7] and its technical applications.

In superconductors, electrons are bound in Cooper pairs, which usually have a singlet structure and therefore carry only charge but no spin. The quasiparticle excitations, however, may carry both charge and spin. Non-equilibrium charge transport in superconductors has been investigated intensely in the 1970s and 1980s, mostly in the vicinity of the critical temperature [8–10] and more recently also in the low-temperature regime [11–13]. In contrast, only few experiments on quasiparticle spin transport [14] have been reported, and the subject remains poorly understood. For example, both anomalously short [15] and anomalously long [16] spin relaxation times have been reported in superconducting aluminum.

In this paper, we summarize some of our recent experimental results on non-equilibrium charge and spin transport in nanoscale superconductors [12,17–18], and perform additional numerical analysis to obtain more insight into the physical mechanisms.

## Results and Discussion

**Figure 1:**
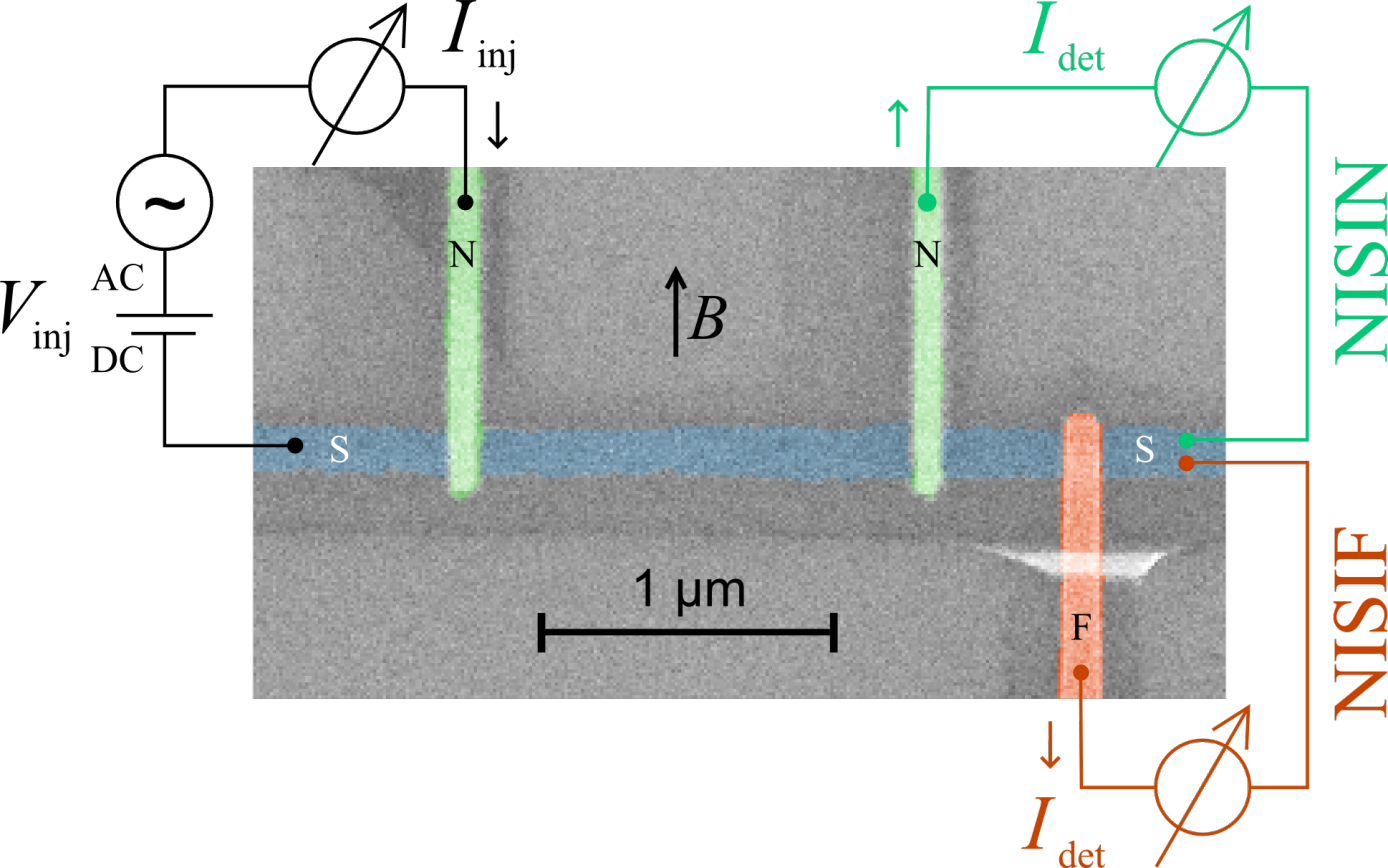
False color scanning electron microscopy image of one of our samples, together with the measurement scheme. The samples consist of a central superconducting wire (S), with normal-metal (N) and/or ferromagnetic (F) wires attached to it via tunnel contacts [18].

Figure 1 shows a typical sample layout and measurement scheme. A central superconducting aluminum wire is contacted by several normal-metal (copper) or ferromagnetic (iron) electrodes attached via thin tunnel barriers. A dc bias voltage *V*_inj_ with a small superimposed low-frequency ac excitation is applied to one junction (injector), and the resulting current *I*_inj_ flowing into the junction is measured to determine the local differential conductance *g*_loc_ = *dI*_inj_/*dV*_inj_. Simultaneously, the current *I*_det_ flowing out of a nearby detector junction is measured to obtain the nonlocal conductance *g*_nl_ = *dI*_det_/*dV*_inj_. The nonlocal conductance was measured for different contact distances *d*, and different material combinations, for which both injector and detector could be either normal (N) or ferromagnetic (F). These configurations will be labeled by *A*ISI*B*, where *A* and *B* denote the injector and detector contacts, respectively. Two examples (NISIN and NISIF) are indicated in Figure 1. The measurements were carried out in a dilution refrigerator at temperatures down to about 50 mK, and with a magnetic field *B* applied along the substrate plane parallel to the copper or iron wires. The thickness of the aluminum films was *t*_Al_ = 12–30 nm, and for the thinnest films, critical fields exceeding 2 T were observed.

**Figure 2:**
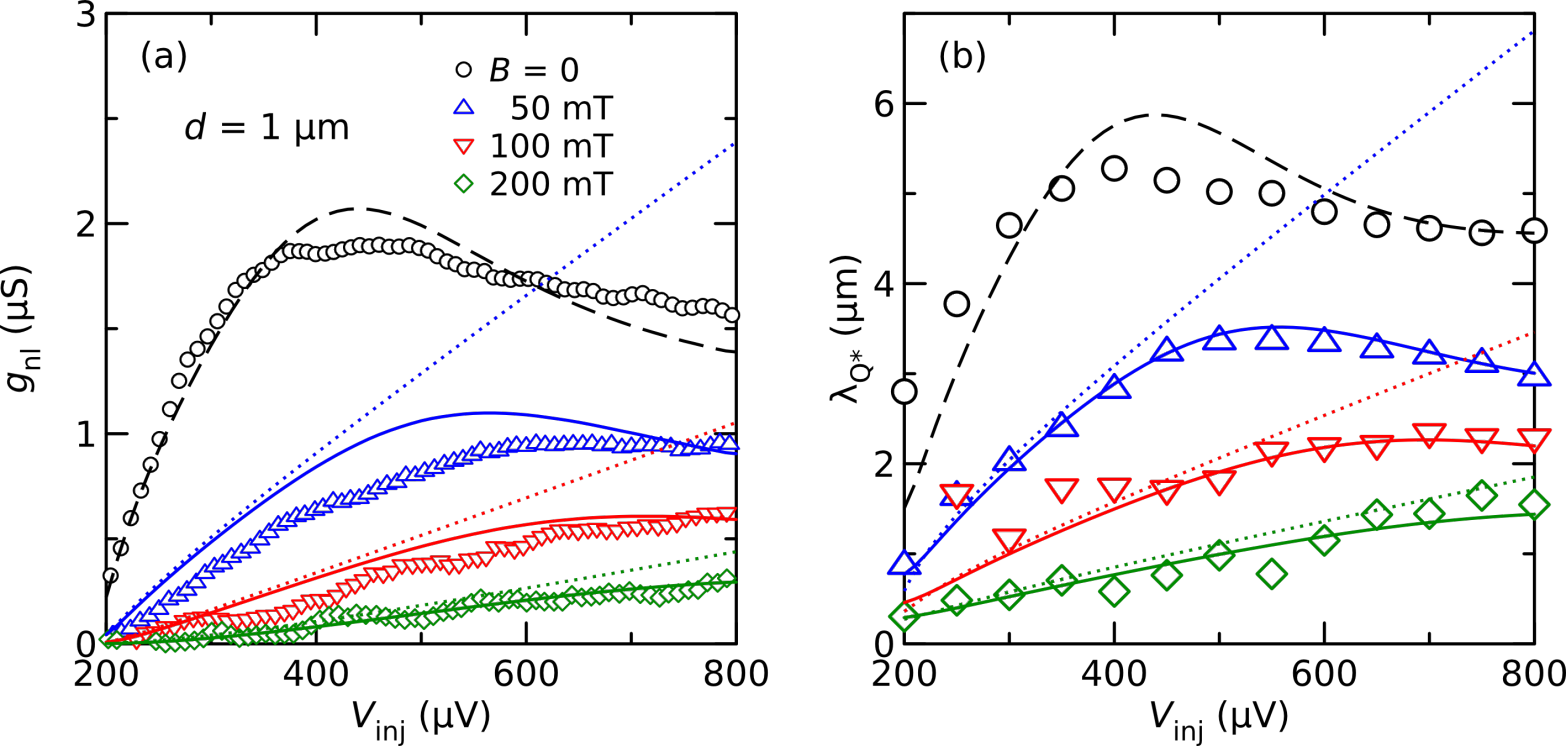
(a) Nonlocal conductance of one contact pair of an NISIN sample with *d* = 1 μm as a function of the injector bias *V*_inj_ for different magnetic fields *B*. (b) Charge imbalance relaxation length 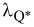. Data taken from [12], the lines are various model predictions explained in the text.

Before we discuss the spin signal observed by using ferromagnetic detector junctions, we analyze the charge imbalance signal observed in an NISIN configuration. The aluminum film thickness of this sample was *t*_Al_ = 30 nm, with a critical field *B*_c_ = 0.53 T. Here, the effect of the applied field is mostly orbital pair breaking, and the Zeeman splitting of the density of states does not play a significant role. In Figure 2a, we show the nonlocal conductance *g*_nl_ of a pair of contacts at low temperature and for bias voltages above the energy gap Δ ≈ 200 μeV of the superconductor. By fitting *g*_nl_ at a given bias voltage for different contact distances to an exponential decay, we can obtain a bias-dependent charge relaxation length 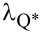 (see [12] for details). The corresponding results are shown in Figure 2b.

Since we are interested here mostly in the behavior at finite magnetic fields, where Green’s function methods are most appropriate, we model the data with the linearized kinetic equation derived by Schmid et al. [19]. A simple analytical approximation that neglects the cooling of the quasiparticles (see Supporting Information File 1) yields the charge-imbalance relaxation length at low temperature

[1]
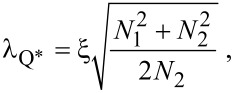


where *N*_1_ is the density of states in the superconductor, *N*_2_ is the real part of the anomalous Green’s function, 
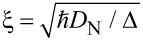
 is the dirty-limit coherence length, and *D*_N_ is the normal-state diffusion coefficient. The nonlocal conductance due to charge imbalance within the same approximation is

[2]
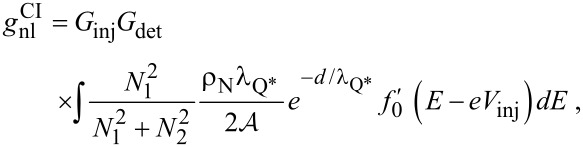


where 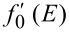 is the derivative of the Fermi function, *ρ*_N_ is the normal-state resistivity of the superconductor, and 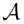 is the cross-section area of the superconducting wire.

In Figure 2, we compare the model predictions to the experimental data. We proceeded by first fitting 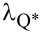 at finite magnetic fields with the simple “no-cooling” approximation Equation 1. Here, we assume that the pair-breaking strength follows the relation ζ = (*B*/*B*_c_)^2^/2 for a magnetic field applied parallel to a thin film, and use the diffusion coefficient *D*_N_ as the single free fit parameter for all curves. These fits are shown as dotted lines in Figure 2b. As can be seen, a good fit can be made for the initial slope of the data, and we obtain *D*_N_ = 70 cm^2^/s from the fit, a value somewhat larger than the independent estimate (40 cm^2^/s) from the resistivity. Without additional fitting, we can then plot the predictions for the nonlocal conductance according to Equation 2 in Figure 2a. For large bias, the experimental data (both *g*_nl_ and 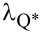) deviate downward from the fits. Full numerical simulations that include cooling, with the characteristic inelastic scattering time τ_E_ as the only remaining fit parameter, are shown as solid lines in Figure 2. Excellent agreement with the experimental data for 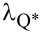 can be achieved for τ_E_ = 12 ns. The agreement for the nonlocal conductance is not as good as for 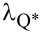, but still satisfactory. We finally attempted to fit the data at zero field, i.e., for ζ = 0. The predictions exceeded the experimental data by about a factor of two, both for *g*_nl_ and 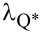 (not shown). We attribute this discrepancy to the fact that at zero applied field, any small additional source of pair breaking, such as gap anisotropy, magnetic impurities, spatial profile of the gap due to quasiparticle injection, etc., may contribute to charge relaxation [20]. A reasonable fit (dashed lines) could be obtained by setting ζ = 8 × 10^−4^ to account for all these pair-breaking perturbations. At zero field, we find a relaxation length of a few micrometers, which corresponds to characteristic time scales of a few nanoseconds. Recently, some experiments reported shorter time scales (sometimes by orders of magnitude) under similar conditions [21–22]. In contrast, our results are quantitatively consistent with the “old” knowledge obtained from experiments close to the critical temperature [23–25], as well as more recent low-temperature experiments on the spatial decay of charge imbalance in thin wires [11,13]. Both experimentally and theoretically, we find that the charge relaxation length decreases with increasing magnetic field, and is smallest at energies just above the gap. This is the parameter range where the spin signal is observed by the ferromagnetic detectors described below. Also, in this parameter regime we can use the analytical “no-cooling” approximation (Equation 2) to describe the charge imbalance.

**Figure 3:**
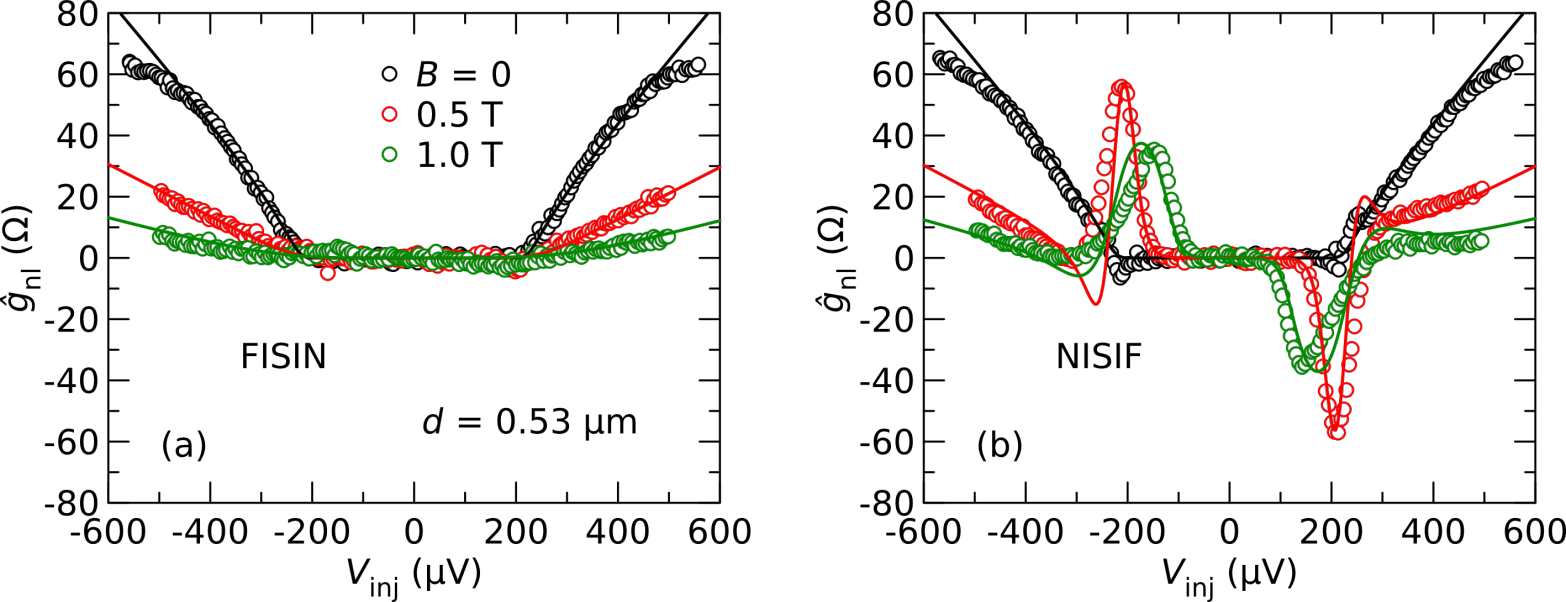
Normalized nonlocal conductance of one contact pair in an FISIN (a) and NISIF (b) configuration as a function of *V*_inj_ for different magnetic fields *B*. Symbols are experimental data [18], lines are fits explained in the text.

In Figure 3 we compare the nonlocal conductance for an FISIN (a) and NISIF (b) configuration, while using the same pair of contacts, but reversing the roles of injector and detector. We plot here the normalized nonlocal conductance 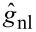 = *g*_nl_/*G*_inj_*G_det_*, where *G*_inj_ and *G_det_* are the normal-state conductances of the injector and detector junctions, respectively. In the FISIN configuration, the nonlocal conductance is negligible at bias voltages below the gap. At bias voltages above the gap, the signal initially increases almost linearly, and then the slope decreases except for the highest magnetic fields. The signal is an even function of the bias and can be attributed to charge imbalance, as described above, since the normal-metal detector is not sensitive to spin accumulation. The lines are fits to Equation 2.

For the NISIF configuration, shown in Figure 3b, a similar signal is observed at *B* = 0. Upon increasing the field, however, two additional peaks appear near the gap edge, with opposite sign for opposite bias polarity. These features can be attributed to spin injection into the Zeeman-split density of states of the superconductor [17–18,22,26], which is probed by the ferromagnetic detector in this configuration. Spin-polarized tunneling can be described by two independent conductances *g*_↓_ and *g*_↑_ for the two spin orientations. The conductance is then given by the sum *g*_↓_ + *g*_↑_, whereas the spin current is proportional to the difference *g*_↓_ − *g*_↑_. The lines in Figure 3b are the sum of the charge-imbalance contribution shown in Figure 3a and an additional contribution 
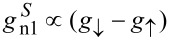
 to account for the spin signal. For the latter, we use parameters that we obtained from fits of the local conductance of the injector junctions, which leaves only the overall signal amplitude as a free fit parameter. As can be seen, the reasonable fit can be obtained over the entire bias range.

**Figure 4:**
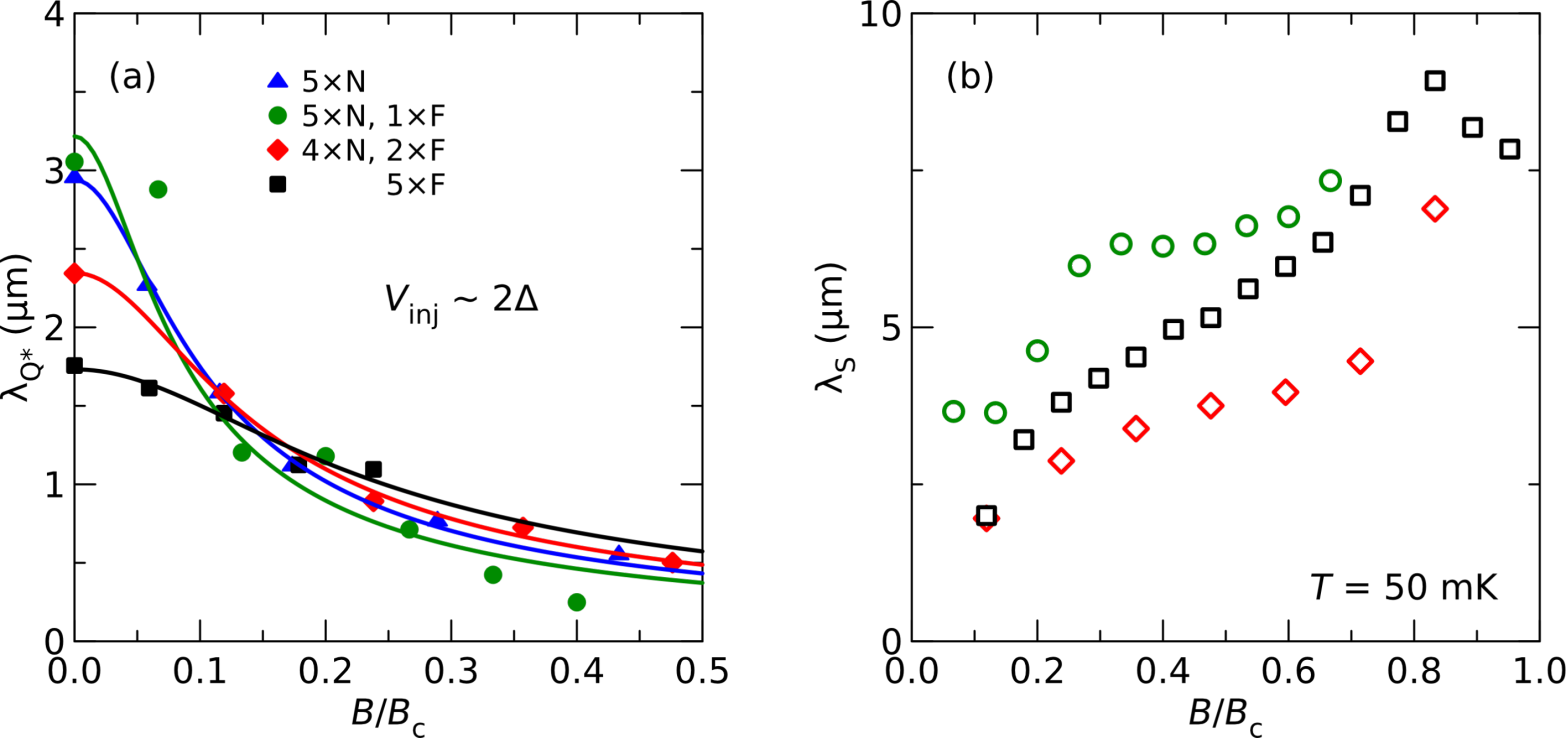
Charge relaxation length 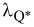 at a bias voltage of about 2Δ (a) and spin diffusion length *λ_S_* (b) for different samples as a function of normalized magnetic field *B*/*B*_c_. The samples have different number of ferromagnetic (F) and normal-metal (N) contacts, as indicated in the legend. Symbols are experimental data [17–18], lines are fits explained in the text.

In Figure 4, we compare the lengths of charge and spin relaxation of several samples with similar properties of the aluminum film as a function of the normalized magnetic field *B*/*B*_c_. The samples have different numbers of ferromagnetic and normal-metal junctions, as indicated in the figure. In Figure 4a, we plot the charge relaxation length 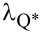 obtained at a bias voltage of about 2Δ, for which 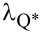 is usually largest at zero field (compare Figure 2). 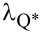 is typically a few micrometers at zero field, and then quickly drops. The lines are fits to Equation 1. The spin relaxation length λ_S_ is found by fitting the area *A* of the spin-signal peaks as a function of contact distance to an exponential decay [17–18]. At small fields, λ_S_ is similar to 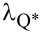, but then strongly increases with increasing field. At present, no theoretical model for high-field spin diffusion and relaxation in superconductors is available, therefore only a tentative interpretation is possible. The normal-state spin diffusion length in the samples is typically less than 500 nm, which means a tenfold increase in the superconducting state. A possible relaxation mechanism could be a two-stage process of spin-flip scattering and recombination, which has been considered theoretically in a different context [27–28]. A generalization of existing models for the non-equilibrium transport in superconductors [19,29] to the case of large Zeeman splitting, treating both charge and spin degrees of freedom on an equal footing, would be highly desirable.

**Figure 5:**
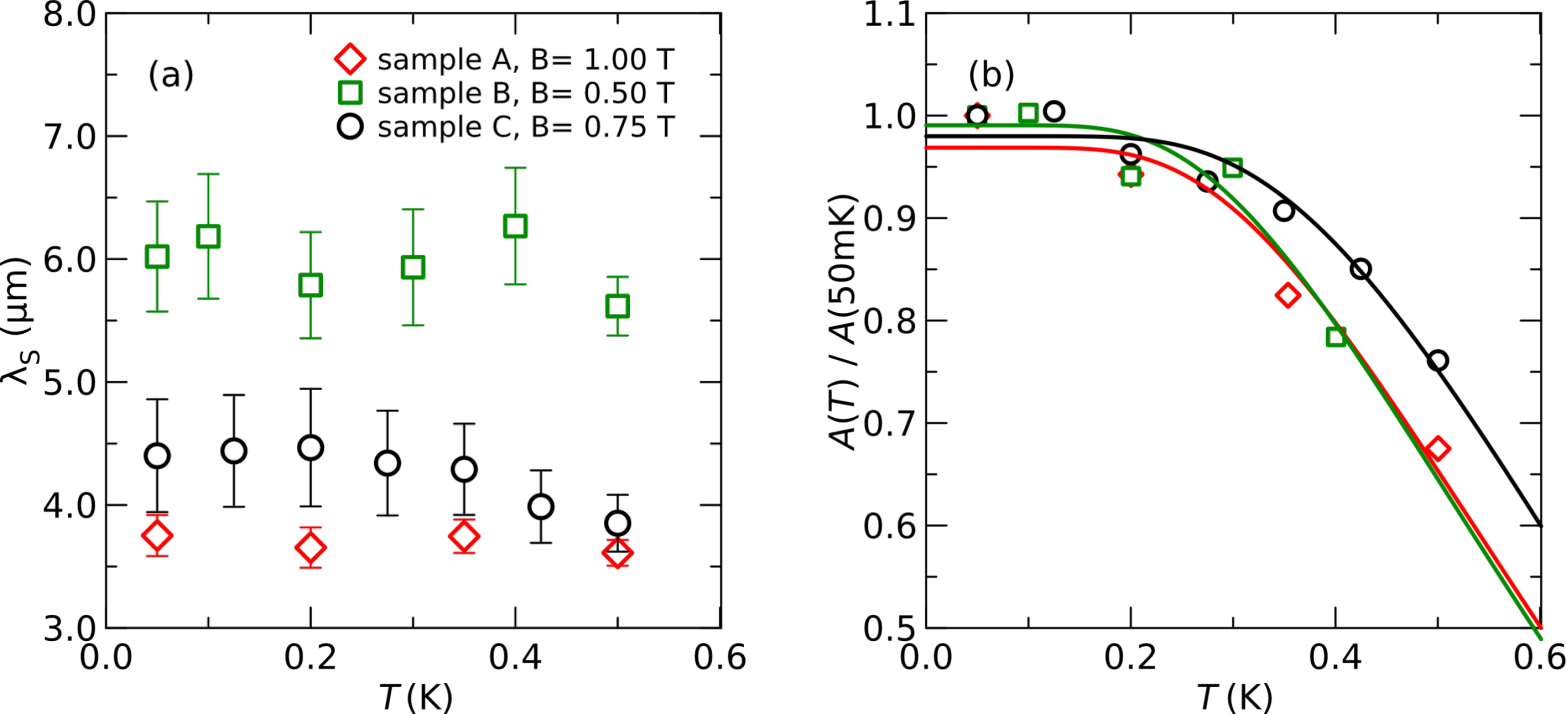
Spin relaxation length λ_S_ (a) and amplitude *A* of the spin signal (b) for different samples as a function of the temperature *T*. Symbols are experimental data [17–18], lines are fits explained in the text.

Figure 5 shows the evolution of the spin relaxation length λ_S_ and the amplitude *A* of the spin signal as a function of the temperature. λ_S_ is independent of the temperature within the accuracy of the experiment, similar to 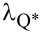 in the same temperature range [12]. In contrast, the signal amplitude decreases with increasing temperature. The spin-injection rate proportional to *g*_↓_ − *g*_↑_ inferred from the local conductance does not change appreciably in this temperature range, except for thermal broadening, which should not affect the overall peak area *A*. Thus, since neither injection nor relaxation cause the signal change, the decrease of signal amplitude must be related to the detection process. A simple model based on the tunnel Hamiltonian yields [17]

[3]
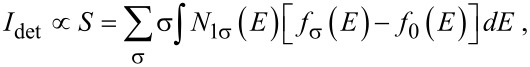


where *S* is the net spin accumulation, *f*_σ_(*E*) is the quasiparticle distribution for spin σ in the superconductor, and *f*_0_ denotes the Fermi distribution in the ferromagnetic detector junction. As can be seen, the detector signal is proportional to the difference of the distribution functions in the superconductor and ferromagnet. The former is determined by spin injection, whereas the latter can be assumed to be (nearly) at equilibrium at the bath temperature. Therefore, we can expect the spin signal to decrease as the bath temperature is raised. A very simple model to describe this drop can be obtained by assuming that non-equilibrium injection raises the effective temperature of the quasiparticles inside the superconductor to about 1 K, as we have found in similar structures with normal-metal junctions [30], and that most quasiparticles have an energy close to the energy gap *E*_g_, which is typically around 0.5–0.75 × Δ_0_ at the fields of the experiments. Then, the spin signal should be proportional to *f*_0_(*E*_g_, 1 K) − *f*_0_(*E*_g_, *T*). Fits to this model are shown in Figure 5b. As can be seen, the agreement is quite good, despite the oversimplification of the model. We note that, usually, the current through an NIS junction does not depend on the temperature of the normal metal due to particle–hole symmetry. This is no longer true if a spin-dependent density of states in the superconductor is combined with a spin-dependent tunnel conductance, as it is the case in our experiment. For this case, large thermoelectric effects driven by the temperature difference between superconductor and ferromagnet have been predicted recently [31–32].

## Conclusion

We have presented an analysis of our recent experiments on spin and charge transport in nanoscale superconductors at very low temperatures and high magnetic fields. We find that charge imbalance can be described surprisingly well with existing models, despite the fact that they were initially developed for experiments close to the critical temperature. Charge relaxation is very fast at energies just above the gap. This is the bias regime, in which we observe long-range spin transport in the presence of a Zeeman splitting of the density of states. By comparing the relaxation lengths for charge and spin, we can conclude that spin currents in this regime are nearly chargeless. While no detailed model of spin transport and relaxation is available yet, we find that simple models based on the tunnel Hamiltonian explain the dependence of spin injection and detection on bias, magnetic field and temperature. The ability to create and transport pure spin currents in superconductors may be useful for future superconducting spintronics devices. Further, our analysis of the temperature dependence hints at the importance of new thermoelectric effects in nanoscale superconductor-ferromagnet hybrids.

## Supporting Information

File 1 Details of the theoretical model.
