# Supplementary material for: Charge and spin transport in mesoscopic superconductors
Source: Beilstein J Nanotechnol. 2014 Feb 17;5:180–5. doi: 10.3762/bjnano.5.18 (PMC3944028; doi:10.3762/bjnano.5.18)
Supplement: File 1 — Details of the theoretical model. [file Beilstein_J_Nanotechnol-05-180-s001.pdf]

Supporting Information

for

Charge and spin transport in mesoscopic superconductors

Michael J. Wolf, Florian Hübler, Stefan Kolenda and Detlef Beckmann*

Address: Karlsruher Institut für Technologie (KIT), Institut für Nanotechnologie, P.O. Box 3640, D-72021 Karlsruhe, Germany

Email: D. Beckmann - detlef.beckmann@kit.edu

* Corresponding author

Details of the theoretical model

Spectral properties

To obtain the spectral properties of the superconductor needed to describe our experiments, we solve the Usadel equation [1,2], including all the perturbations needed for our purpose. Pair breaking is included by the dimensionless pair-breaking strength

$$\zeta = \frac{1}{2}\left(\frac{B}{B_{\mathrm{c}}}\right)^2 \tag{1}$$

for a thin film with a magnetic field B applied parallel to the film plane, with the critical field B_{c}. It is related to the magnetic-impurity scattering time τ_{s} used in [3] by $\zeta = \hbar/2\Delta_0\tau_{\mathrm{s}}$, where Δ_0 is the superconducting pair potential at $T = 0$ and $B = 0$. The quasiparticle life time due to electron-phonon scattering is included by adding a small imaginary part (Dynes parameter [4]) $\Gamma = \hbar/2\tau_E$ to the energy, where τ_E is the inelastic scattering time (see below). We also include the Zeeman energy $\pm\mu_{\mathrm{B}}B$, and spin-orbit scattering with scattering strength $b_{\mathrm{so}} = \hbar/3\Delta\tau_{\mathrm{so}}$. Since all our junctions have tunnel barriers, we neglect the proximity effect, and assume that the spectral properties of our superconducting wires are independent of position, i.e., we neglect gradient terms in the Usadel equation. The complete Usadel equation with all terms included can be found in [5].

Kinetic equation for charge imbalance

We describe charge imbalance using the kinetic equation derived by Schmid et al. [3]. To apply the kinetic equation to our experiments, we consider a superconducting wire of cross-section $\mathscr{A}$ and normal-state diffusion coefficient D_{N} along the x-axis, an injector junction with normal-state conductance G_{inj} placed at $x = 0$, and a detector junction with normal-state conductance G_{det} placed at $x = d$. We assume that both the wire cross-section and the junction width are small compared to the charge-imbalance relaxation length. We are interested only in the stationary case. The kinetic equation for the transverse-mode distribution function f_{T} then reads

$$D_{\mathrm{N}}M_{\mathrm{T}}(E,E)\frac{d^2}{dx^2}f_{\mathrm{T}}(E) + K_{\mathrm{T}}(E,\{f_{\mathrm{T}}\}) + Q_{\mathrm{T}}(E) + P_{\mathrm{T}}(E) = 0 \tag{2}$$

Here, $M_{\rm T}(E,E') = N_1(E)N_1(E') + N_2(E)N_2(E')$, and N_1 and N_2 are the real parts of the normal and anomalous Green's function found solving the Usadel equation (N_1 is the density of states). The collision integral describing energy relaxation is

$$\begin{aligned} K_{\rm T}(E,\{f_{\rm T}\}) &= -\int dE' \frac{\mu(E-E')M_{\rm T}(E,E')}{\cosh(E/2k_{\rm B}T)\cosh(E'/2k_{\rm B}T)\sinh((E-E')/2k_{\rm B}T)} \\ &\times \left[\cosh^2\left(\frac{E}{2k_{\rm B}T}\right) f_{\rm T}(E) - \cosh^2\left(\frac{E'}{2k_{\rm B}T}\right) f_{\rm T}(E')\right] \end{aligned}$$

where

$$\mu(E) = \frac{\mathrm{sign}(E)E^2}{14\zeta(3)(k_{\rm B}T_{\rm c})^3\tau_E}$$

within the Debye model, and τ_E is the inelastic scattering time for electrons at the Fermi surface in the normal state at $T_{\rm c}$. Charge relaxation is given by

$$Q_{\rm T}(E) = -2\frac{\Delta}{\hbar}N_2(E)f_{\rm T}(E),$$

and the injection rate is

$$\begin{aligned} P_{\rm T}(E) &= \frac{G_{\rm inj}}{2N_0\Omega e^2}N_1(E)f_{\rm inj}(E,V) \\ f_{\rm inj}(E,V) &= \frac{1}{4}\left[\tanh\left(\frac{E+eV}{2k_{\rm B}T}\right) - \tanh\left(\frac{E-eV}{2k_{\rm B}T}\right)\right] \end{aligned}$$

where Ω is the injection volume and N_0 is the density of states of the superconductor per spin. The current flowing from S to N through the detector junction held at zero bias voltage is given by

$$I_{\rm det} = \frac{G_{\rm det}}{e}\int dE N_1(E)f_{\rm T}(E). \tag{3}$$

Approximate analytical solution (without cooling)

At low temperatures, inelastic scattering is expected to freeze out. To obtain a simple analytical solution for $T \to 0$, we therefore neglect the collision integral, i.e., the cooling of the quasiparticles. We also assume that the injector junction is infinitesimally small, with inverse injection volume $\Omega^{-1} = \mathscr{A}^{-1}\delta(x)$. Then the kinetic equation can be easily solved by inserting the Ansatz

$$f_{\mathrm{T}}(x,E) = a(E)e^{-|x|/\lambda_{\mathrm{Q}^*}(E)}, \tag{4}$$

which yields the two conditions

$$\lambda_{\mathrm{Q}^*}(E) = \xi\sqrt{\frac{N_1^2+N_2^2}{2N_2}} \tag{5}$$

$$a(E) = G_{\mathrm{inj}}\frac{\rho_{\mathrm{N}}\lambda_{\mathrm{Q}^*}}{2\mathscr{A}}\frac{N_1}{N_1^2+N_2^2}f_{\mathrm{inj}}(E,V) \tag{6}$$

where we have introduced the dirty-limit coherence length $\xi = \sqrt{\hbar D_{\mathrm{N}}/\Delta}$ and the normal-state resistivity of the superconductor $\rho_{\mathrm{N}} = (2N_0e^2D_{\mathrm{N}})^{-1}$. Inserting this solution into (3) yields the detector current

$$I_{\mathrm{det}} = \frac{G_{\mathrm{inj}}G_{\mathrm{det}}}{e}\int dE\frac{\rho_{\mathrm{N}}\lambda_{\mathrm{Q}^*}}{2\mathscr{A}}\frac{N_1^2}{N_1^2+N_2^2}e^{-d/\lambda_{\mathrm{Q}^*}}f_{\mathrm{inj}}(E,V) \tag{7}$$

Using the symmetry properties of the various quantities, we finally obtain

$$g_{\mathrm{nl}} = \frac{dI_{\mathrm{det}}}{dV_{\mathrm{inj}}} = G_{\mathrm{inj}}G_{\mathrm{det}}\int\frac{N_1^2}{N_1^2+N_2^2}\frac{\rho_{\mathrm{N}}\lambda_{\mathrm{Q}^*}}{2\mathscr{A}}e^{-d/\lambda_{\mathrm{Q}^*}}f'(E-eV_{\mathrm{inj}})dE, \tag{8}$$

where $f'(E) = \cosh^{-2}(E/2k_{\mathrm{B}}T)/4k_{\mathrm{B}}T$ is the derivative of the Fermi function.

Numerical solution

For a full numerical solution, we discretize the equation on grid points E_i and x_k. The kinetic equation (2) then turns into a linear equation system for the distribution function $f_{ik} = f_{\mathrm{T}}(x_k,E_i)$.

This equation system is solved by standard library routines [6]. The size of the grid points is chosen small enough to not affect the results ($\delta E \approx 10$ μeV, $\delta x = 500$ nm). From the numerical solution, we obtain the nonlocal conductance as a function of bias and contact distance, and analyze it in the same way as the experimental data [7] to obtain the relaxation length.

Spin imbalance

In order to describe the local conductance of the injector junctions, we use the theory of tunneling in superconductors in high magnetic field [8,9]. The contribution of a single spin projection $\sigma = \pm 1$ to the tunnel conductance is given by

$$g_\sigma = \frac{G_{\text{inj}}}{2}\left(1 - \sigma P_{\text{inj}}\right)\int N_{1\sigma}(E) f' dE, \tag{9}$$

where P_{inj} is the spin polarization of the tunnel conductance, and $N_{1\sigma}(E)$ is the density of states in the superconductor for spin projection σ, obtained by solving the Usadel equation. The injector conductance is given by the sum of the two spin contributions,

$$g_{\text{loc}} = g_\downarrow + g_\uparrow, \tag{10}$$

whereas the differential spin current

$$\frac{dI_\sigma}{dV_{\text{inj}}} \propto g_\downarrow - g_\uparrow \tag{11}$$

is proportional to their difference. From fits of the local conductance, we can therefore infer the bias-dependent spin injection rate. A simple tunnel Hamiltonian model for the spin-related contribution to the detector current yields [10,11]

$$I_{\text{det}}^S = \frac{G_{\text{det}} P_{\text{det}}}{2e} \sum_\sigma \sigma \int N_{1\sigma}(E)\left[f_\sigma(E) - f_0(E)\right] dE, \tag{12}$$

where $f_\sigma(E)$ is the quasiparticle distribution for spin σ in the superconductor, and f_0 denotes the Fermi distribution in the ferromagnetic detector junction. Combining (11) and (12), we expect the contribution of spin accumulation to the nonlocal conductance to be

$$g_{\mathrm{nl}}^{S} \propto P_{\mathrm{det}} \left(g_{\downarrow} - g_{\uparrow}\right) . \qquad (13)$$

References

1. Usadel, K. D. *Phys. Rev. Lett.* **1970**, *25*, 507.

2. Belzig, W.; Wilhelm, F. K.; Bruder, C.; Schön, G.; Zaikin, A. D. *Superlatt. Microstruct.* **1999**, *25*, 1251.

3. Schmid, A.; Schön, G. *J. Low Temp. Phys.* **1975**, *20*, 207.

4. Dynes, R. C.; Narayanamurti, V.; Garno, J. P. *Phys. Rev. Lett.* **1978**, *41*, 1509.

5. Alexander, J. A. X.; Orlando, T. P.; Rainer, D.; Tedrow, P. M. *Phys. Rev. B* **1985**, *31*, 5811.

6. Anderson, E.; Bai, Z.; Bischof, C.; Blackford, S.; Demmel, J.; Dongarra, J.; Du Croz, J.; Greenbaum, A.; Hammarling, S.; McKenney, A.; Sorensen, D. *LAPACK Users' Guide*, 3rd ed.; Society for Industrial and Applied Mathematics: Philadelphia, PA, 1999.

7. Hübler, F.; Camirand Lemyre, J.; Beckmann, D.; v. Löhneysen, H. *Phys. Rev. B* **2010**, *81*, 184524.

8. Maki, K. *Prog. Theor. Phys.* **1964**, *32*, 29.

9. Meservey, R.; Tedrow, P. M. *Phys. Rep.* **1994**, *238*, 173.

10. Zhao, H. L.; Hershfield, S. *Phys. Rev. B* **1995**, *52*, 3632.

11. Hübler, F.; Wolf, M. J.; Beckmann, D.; v. Löhneysen, H. *Phys. Rev. Lett.* **2012**, *109*, 207001.
